# Collateral status predicts functional outcome in early-treated large-core anterior circulation stroke

**DOI:** 10.3389/fstro.2026.1755828

**Published:** 2026-04-14

**Authors:** Andrés Gallardo, Pablo M. Lavados, Pablo Albiña-Palmarola, Gabriel Cavada, Andrés Roldán, Verónica V. Olavarría

**Affiliations:** 1 Department of Neurology, Hospital Base Valdivia, Valdivia, Chile; 2 Department of Neurology and Psychiatry, Clínica Alemana – Universidad del Desarrollo, Santiago, Chile; 3 Neuroradiologische Klinik, Kopf- und Neurozentrum, Klinikum Stuttgart, Stuttgart, Germany; 4 Faculty of Medicine, School of Public Health, University of Chile, Santiago, Chile

**Keywords:** acute ischemic stroke, collaterals, CTA, large core, thrombectomy

## Abstract

**Background and purpose:**

Endovascular therapy (EVT) is increasingly offered to patients with large-core acute ischemic stroke (AIS), yet outcomes remain highly heterogeneous. Collateral circulation may be a key determinant of infarct evolution and recovery, but its role in early-window large-core stroke is not fully defined.

**Methods:**

We retrospectively analyzed consecutive adults from a prospective stroke registry who presented within 6 h with anterior-circulation large-vessel occlusion, NIHSS ≥6, and a large ischemic core (MRI core >50 mL or CT perfusion core >70 mL, up to 150 mL). All patients received reperfusion therapy (intravenous thrombolysis, EVT, or both). Collateral status on baseline single-phase CTA was graded using the Tan scale (0–3); no patients had grade 3. The primary outcome was 90-day modified Rankin Scale (mRS); secondary outcome was NIHSS at discharge.

**Results:**

Fifty-four patients met inclusion criteria (Tan 0: *n* = 24; Tan 1: *n* = 14; Tan 2: *n* = 16). Baseline NIHSS, ASPECTS, and core volume were similar across groups. Patients without collaterals (Tan 0) had worse 90-day outcomes (median mRS 4 [IQR 3–6]) compared with those with Tan 1 (2 [IQR 1–3]) or Tan 2 (1 [IQR 1–2]) collaterals (both p < 0.001), whereas Tan 1 and Tan 2 did not differ significantly (*p* = 0.27). NIHSS at discharge showed a similar gradient. In proportional-odds logistic regression, each one-grade increase in collateral status was associated with lower odds of worse 90-day mRS (adjusted per-grade OR 0.32; 95% CI 0.15–0.68; *p* = 0.003).

**Conclusion:**

In early-treated large-core AIS, even simple CTA-based collateral assessment strongly predicts recovery. Patients with absent collaterals follow a distinctly poorer trajectory, while those with any collateral filling behave more favorably. Incorporating collateral status into routine evaluation may improve prognostic accuracy and support treatment decisions in this challenging subgroup.

## Introduction

Endovascular therapy (EVT) has revolutionized the management of acute ischemic stroke (AIS) due to large-vessel occlusion (LVO), with multiple randomized controlled trials (RCTs) and subsequent meta-analyses demonstrating substantial benefit in selected patients (Goyal et al., 2016; Alves et al., 2025; Saver et al., 2016). Early landmark trials and the HERMES collaboration meta-analysis established EVT as the standard of care for patients with small-to-moderate core infarcts, showing improved rates of functional independence compared to medical management alone (Goyal et al., 2016; Alves et al., 2025). However, the benefit of EVT in patients with large ischemic cores has been historically uncertain, since most pivotal trials excluded patients with ASPECTS ≤ 5 or core volumes >70 mL (Lin et al., 2025; Campbell et al., 2019).

Recent evidence has begun to address this gap. The RESCUE-Japan LIMIT trial defining large ischemic core as ASPECTS 3–5 or core ≥50 mL—first demonstrated that EVT can improve outcomes in this population (Yoshimura et al., 2022). ANGEL-ASPECT and SELECT2 subsequently reinforced this benefit across broader core volumes, extending eligibility up to 150 mL (Huo et al., 2023; Sarraj et al., 2023). More recently, randomized trials including TESLA, LASTE and TENSION have further strengthened this signal, reinforcing the benefit of EVT across heterogeneous imaging criteria and time windows (Lin et al., 2025). Taken together, these studies show a coherent pattern in which EVT confers benefit across a wide spectrum of large-core presentations, although functional independence rates remain lower than in earlier HERMES cohorts (Goyal et al., 2016).

Collateral circulation has emerged as a critical determinant of prognosis in AIS, influencing infarct progression, penumbral viability, and response to reperfusion therapies (Alves et al., 2025; Jansen et al., 2019; Jung et al., 2017; Olthuis et al., 2023). Imaging studies using CTA and multiphase CTA have shown that collateral status is strongly associated with functional outcomes after EVT (Alves et al., 2025; Jansen et al., 2019; Menon et al., 2015). Earlier studies have shown that collaterals can shape how an ischemic stroke evolves. When some flow is maintained through these alternative pathways, the damaged area usually grows more slowly and patients often remain more stable in the first hours (Broocks et al., 2020; Jung et al., 2017). But when collaterals are absent, the injury tends to spread quickly, reperfusion is less effective, and neurological worsening is more common (Wu et al., 2025; Broocks et al., 2020).

Beyond EVT, the role of intravenous thrombolysis (IVT) in patients with large-core infarcts and borderline ASPECTS 5–6 remains uncertain. Most pivotal IVT trials excluded this subgroup, and evidence is largely derived from retrospective studies and registries (Busto et al., 2023). Observational data suggest IVT may reduce infarct progression or facilitate early reperfusion prior to EVT, particularly in patients with preserved collaterals, though the risk of hemorrhagic transformation remains significant (Busto et al., 2023; Alves et al., 2025).

Furthermore, collateral status has also emerged as an important factor in studies evaluating treatment beyond the early time window. In DAWN and DEFUSE-3, advanced or multimodal imaging was used to select patients who could still benefit from EVT up to 24 h after symptom onset (Albers et al., 2018; Nogueira et al., 2018). These imaging approaches were largely reserved for the extended window, while early-window practice has moved toward simpler and faster selection strategies. More recently, MR CLEAN-LATE highlighted how much collateral flow can matter, showing that patients with more preserved collaterals were more likely to benefit from EVT beyond 6 h, even though the trial excluded patients with marked early infarct changes on CT. Taken together, these findings reinforce the idea that collateral status can serve as a practical and reliable marker when selecting patients in the late window (Olthuis et al., 2023).

Taken together, current evidence raises the possibility that collateral circulation could influence prognosis and help guide treatment decisions in large-core AIS. However, real-world data remain limited, and further studies are needed to clarify whether and under what conditions collateral-based selection can be integrated into routine clinical practice.

We hypothesized that collateral status assessed on baseline CTA would strongly influence both neurological recovery and long-term functional outcomes, even among patients with similar ASPECTS and core volumes.

Despite the growing body of evidence supporting thrombectomy in large-core stroke, clinical outcomes remain highly variable, suggesting that additional biological factors beyond infarct volume may influence recovery in this population.

## Methods

We retrospectively analyzed a prospective cohort from the Stroke Registry of Clínica Alemana de Santiago (RECCA), including all consecutive adult AIS patients admitted between August 2019 and December 2024.

Inclusion criteria: (1) symptom onset ≤ 6 h; (2) NIHSS ≥6; (3) CTA confirming ICA terminal, M1, or M2 occlusion; (4) Ischemic core was quantified with RAPID software (iSchemaView). Large core was defined as >50 mL on MRI (ADC ≤ 620 × 10^−6^ mm^2^/s) or >70 mL on CT perfusion (CBF < 30%), up to a maximum of 150 mL; (5) Reperfusion therapy (IV thrombolysis plus thrombectomy, direct thrombectomy, or IV thrombolysis alone with tenecteplase in cases of angiographic recanalization).

The initial evaluation included NCCT and single-phase CTA according to institutional protocol. The local workflow allows this initial imaging to be complemented by an abbreviated MRI or CT-perfusion protocol performed after administration of the thrombolytic bolus and once the neurointerventional team has been activated.

Collateral status was graded on baseline CTA using the Tan scale (Tan 0 = absent; Tan 1 = ≤ 50% supply; Tan 2 = >50% but < 100%; Tan 3 = complete). Since no patients presented with Tan grade 3, analyses were limited to grades 0–2. Two independent blinded readers evaluated collateral status using 20-mm MIP reconstructions from single-phase CTA obtained 15–25 s after contrast injection. Interobserver agreement was quantified with the quadratic weighted kappa coefficient. When the two primary readers differed by ≥2 collateral grades, the case was re-reviewed in a consensus session, and the final grade was assigned by joint agreement.

The primary outcome was functional status at 90 days, measured with the modified Rankin Scale (mRS), and the secondary outcome was neurological deficit at discharge, measured with the NIHSS. Statistical analyses were performed using STATA version 16. Continuous variables are presented as median with interquartile range or mean ± standard deviation depending on their distribution, and categorical variables are reported as counts and percentages.

Because the modified Rankin Scale (mRS) is an ordinal outcome, the association between collateral grade and functional outcome was assessed using proportional-odds ordinal logistic regression. This approach evaluates changes across the entire distribution of disability rather than focusing only on dichotomous thresholds such as mRS 0–2.

Given the relatively small sample size, the number of variables included in the adjusted model was intentionally kept limited to reduce the risk of overfitting. Based on clinical relevance, the model was adjusted for the use of intravenous thrombolysis and endovascular therapy.

A two-sided *p* value < 0.05 was considered statistically significant.

## Results

A total of 54 patients met the inclusion criteria, distributed by collateral grade as follows: Grade 0 (*n* = 24), Grade 1 (*n* = 14), and Grade 2 (*n* = 16). Baseline characteristics were broadly comparable across groups (Table 1), with a median NIHSS on admission of 20 [14–26], median ASPECTS of 6 [5–6], and median core volume of 87.9 mL. Collateral status distribution in the cohort included grades 0, 1, and 2, with no patients presenting grade 3 collaterals.

**Table 1:** Baseline characteristics and outcomes of the study cohort (*n* = 54).

Variable	Value
Age, years	68 (57–80)
Female sex	30 (56%)
Admission NIHSS	20 (14–26)
ASPECTS	6 (5–6)
Core volume (mL)	87.9 (74.1–99.9)
Discharge NIHSS	10 (8–12)
90-day mRS	3 (2–4)

Values are reported as median [interquartile range] or n (%). NIHSS, National Institutes of Health Stroke Scale; ASPECTS, Alberta Stroke Program Early CT Score; mRS, modified Rankin Scale.

Functional outcomes at 3 months varied markedly according to collateral status (Table 2). Patients without collaterals had the poorest outcomes, with a median 90-day mRS of 4 (IQR 3–6), compared with 2 (IQR 1–3) in patients with moderate collaterals and 1 (IQR 1–2) in those with good collaterals. *Post-hoc* pairwise comparisons showed that both intermediate and good collaterals were associated with significantly better outcomes than absent collaterals, whereas outcomes did not differ significantly between intermediate and good collateral grades (Table 3). Neurological status at discharge showed a similar pattern (Table 4). Median discharge NIHSS was 14.5 (10–20), 6 (2–11), and 4 (1–6) across collateral grades 0, 1, and 2, respectively. Pairwise comparisons following the Kruskal–Wallis test demonstrated significant differences between grades 0 vs. 1 and 0 vs. 2, but not between grades 1 vs. 2. Interobserver agreement for the Tan collateral scale was very good (quadratic weighted κ = 0.81; 95% CI 0.70–0.92), confirming the reliability of collateral grading between readers; discrepancies were resolved by consensus.

**Table 2:** Clinical outcomes according to collateral grade.

Collateral grade	*n*	NIHSS discharge (median, IQR)	mRS 90 days (median, IQR)
0 (Absent)	24	14.5 (10–20)	4 (3–6)
1 (Moderate)	14	6 (2–11)	2 (1–3)
2 (Good)	16	4 (1–6)	1 (1–2)

Data are reported as median (IQR). Collateral grade was assessed on baseline CTA using the Tan scale (0 = absent, 1 = moderate, 2 = good). NIHSS, National Institutes of Health Stroke Scale; mRS, modified Rankin Scale.

**Table 3:** Pairwise comparisons of 90-day mRS across collateral grades.

Comparison	Mean difference in mRS	*p*-value
Grade 1 vs. 0	−1.7	< 0.001
Grade 2 vs. 0	−2.4	< 0.001
Grade 2 vs. 1	−0.7	0.275

Values represent mean differences in 90-day mRS between collateral grades. Multiple comparisons were adjusted using the Scheffé method. mRS, modified Rankin Scale.

**Table 4:** NIHSS at discharge by collateral grade.

Comparison	Difference in mean ranks	*p*-value
Grade 0 vs. 1	7.2	0.048
Grade 0 vs. 2	10.1	0.010
Grade 1 vs. 2	3.0	0.200

Values represent rank mean differences between collateral groups. Pairwise comparisons were obtained following the Kruskal–Wallis test with multiple comparisons. NIHSS, National Institutes of Health Stroke Scale.

**Figure 1:**
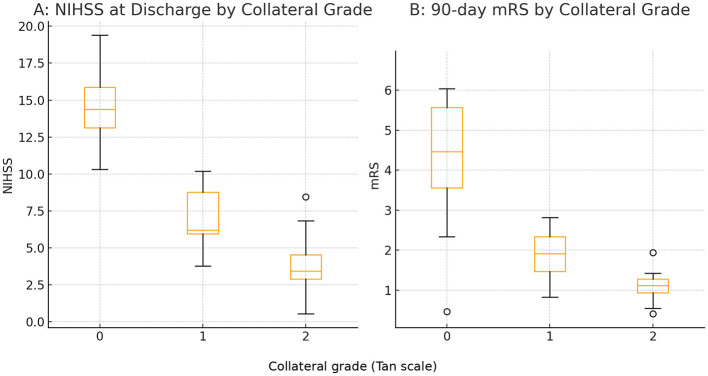
Neurological and functional outcomes according to collateral grade. **(A)** NIHSS score at discharge grouped by collateral grade (0–2). **(B)** Distribution of 90-day mRS by collateral grade. Both neurological status at discharge and 3-month functional outcome improved progressively with better collateral status.

Figure 1 shows the progressive improvement in discharge NIHSS and 90-day mRS across collateral grades. The largest difference was observed between absent and intermediate collaterals, while patients with good collaterals demonstrated an additional, though modest, benefit. Overall, these findings underscore the protective role of collateral circulation in large-core AIS treated within 6 h. Ordinal logistic regression confirmed a strong association between collateral grade and functional outcome. Each one-grade increase in collateral status was associated with lower odds of worse disability at 90 days (adjusted OR 0.32; 95% CI 0.15–0.68; *p* = 0.003).

**Figure 2:**
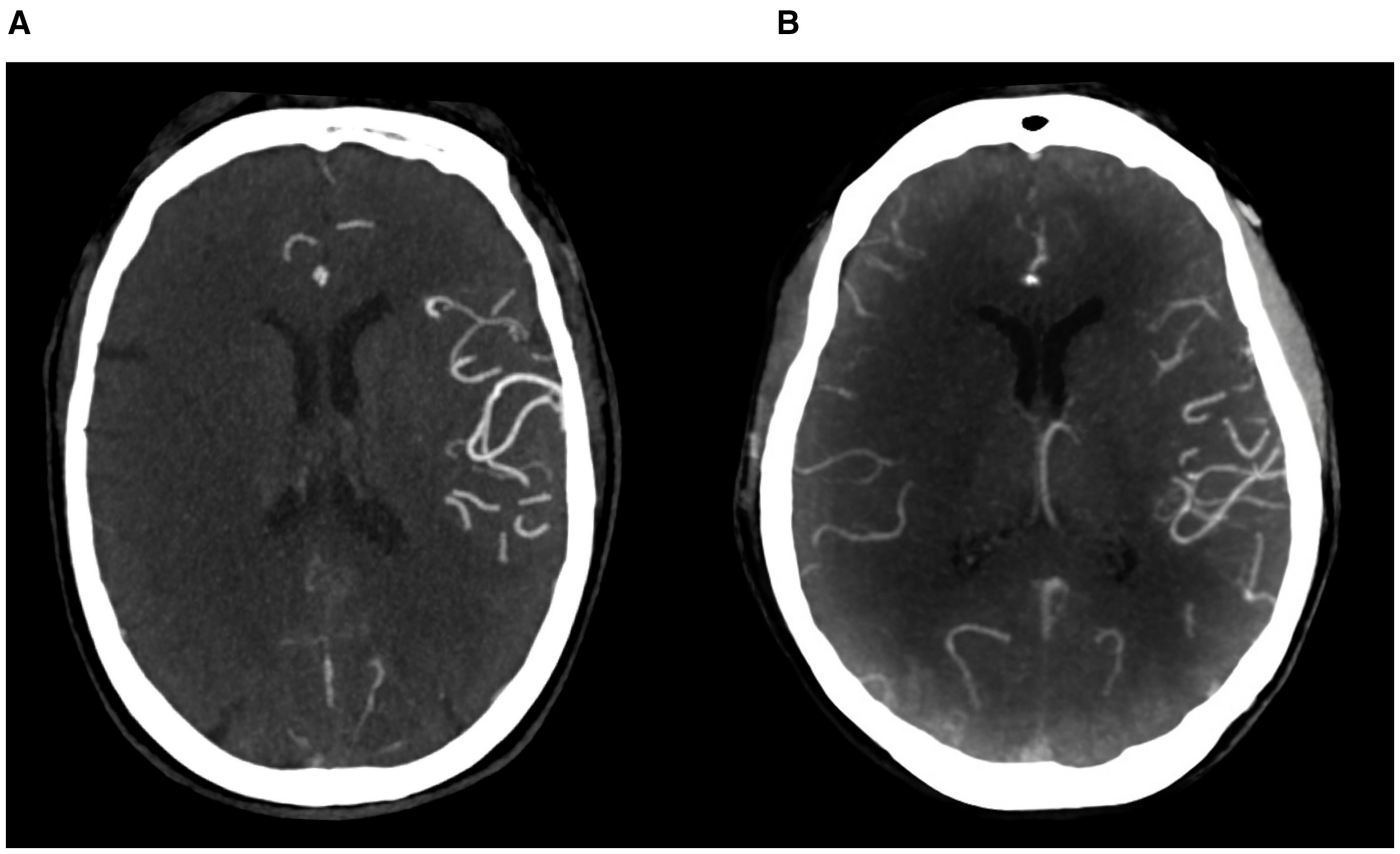
CTA examples of collateral circulation. **(A)** Collateral grade 0, with absent pial arterial filling in the right anterior circulation. **(B)** Collateral grade 2, demonstrating ≥50% pial filling of the ischemic right anterior circulation territory. The comparison highlights the striking clinical and perfusion differences between poor and robust collateral networks.

Collateral distribution in the cohort was heterogeneous, with a predominance of absent or moderate collateral patterns. Representative CTA examples are shown in Figure 2, illustrating the contrast between absent collaterals (grade 0) with no distal pial filling and good collaterals (grade 2) with ≥50% pial filling of the territory. These imaging patterns were consistent with the clinical outcomes observed, as patients with absent collaterals showed markedly worse NIHSS at discharge and 90-day mRS compared with those with good collaterals.

Rates of intravenous thrombolysis (IVT) and endovascular therapy (EVT) were broadly comparable across collateral groups. IVT was administered in 83% of patients with Tan 0 collaterals (20/24), 71% with Tan 1 (10/14), and 56% with Tan 2 (9/16; *p* = 0.12). EVT was performed in 75%, 71%, and 69% of these groups, respectively (*p* = 0.89). Among EVT-treated patients, successful reperfusion (TICI 2b−3) was achieved in 67%, 80%, and 82% of Tan 0, 1, and 2, respectively (χ^2^ = 1.05, *p* = 0.59). Thus, differences in clinical outcomes cannot be attributed to disparities in reperfusion therapies.

## Discussion

In this cohort of patients with early-window large-core acute ischemic stroke, collateral status on baseline CTA emerged as one of the clearest indicators of how patients were likely to evolve. Even though most individuals presented with similarly large ischemic cores, those who demonstrated at least some collateral filling tended to have better recovery at discharge and at 90 days compared with patients without visible collaterals. In this context, where infarct progression is generally rapid even if its velocity varies across patients, this pattern reinforces prior observations indicating that collaterals influence the course of infarct evolution, help preserve hypoperfused yet potentially viable tissue, and shape how this tissue responds once reperfusion is achieved (Jung et al., 2017; Pirson et al., 2021).

The relationship between collateral circulation and outcome likely reflects several biological mechanisms. Collateral flow can slow infarct expansion and maintain some degree of residual perfusion in surrounding tissue, even in patients presenting with large baseline infarcts. Imaging-defined ischemic core may overestimate irreversible injury in the early time window. Residual tissue viability within the apparent core may limit the progression of cytotoxic and vasogenic edema and remain salvageable with reperfusion, potentially reducing the risk of malignant cerebral edema (Sarraj et al., 2025; Xu et al., 2026).

Our findings both align with and help clarify results from recent randomized trials that expanded endovascular therapy to patients with large ischemic cores, including RESCUE-Japan LIMIT (Yoshimura et al., 2022), ANGEL-ASPECT (Huo et al., 2023), and SELECT2 (Sarraj et al., 2023), as well as the more recent ATLAS meta-analysis (Sarraj et al., 2025). Within this evolving evidence landscape, our results highlight a nuance that is rarely emphasized in these studies, namely that even among patients with similar ASPECTS and core volumes, collateral status may help explain why some individuals recover more favorably than others (Broocks et al., 2020; Alves et al., 2025; Jansen et al., 2019; Shin et al., 2014).

As expected in a large-core stroke population, the proportion of patients achieving functional independence (mRS 0–2) was relatively low. However, the ordinal shift analysis suggests that preserved collaterals may still translate into clinically meaningful reductions in disability. Those without collaterals consistently had worse outcomes, whereas patients with moderate or good collaterals tended to behave more similarly to one another. This “all-or-none” pattern has been described before and suggests that in the early time window, simply having some degree of collateral flow may be more important than the exact level of robustness (Jansen et al., 2019; Menon et al., 2015; Liebeskind et al., 2017; Shin et al., 2014). From a clinical perspective, these findings may provide rapid prognostic information in patients with large-core stroke. Importantly, our results should not be interpreted as suggesting that patients with absent collaterals should be excluded from reperfusion therapy. Rather, collateral assessment may help clinicians better understand the expected trajectory of recovery and support more individualized decision-making.

Collateral status may also be relevant when considering reperfusion strategies in large-core stroke (Jung et al., 2017; Christoforidis et al., 2009). However, our study was not designed to evaluate the effect of intravenous thrombolysis according to collateral patterns, and this hypothesis should therefore be interpreted cautiously. Larger prospective studies will be needed to clarify whether collateral status modifies the balance of risks and benefits of thrombolysis in this population.

This discussion becomes particularly relevant in real-world settings where imaging resources and treatment availability vary substantially across regions, especially in the early time window when perfusion imaging is not widely accessible, multimodal protocols may over-exclude patients, and decisions must be made quickly with little time for additional post-processing. Single-phase CTA, interpreted with a simple and reproducible scale such as Tan, allows clinicians to estimate collateral status rapidly and with reasonable reliability (Busto et al., 2023; Alves et al., 2025; Jansen et al., 2019; Menon et al., 2015). In resource-limited settings, this information can guide decisions about endovascular therapy, help prioritize transfers to thrombectomy centers, support thrombolysis decisions, and provide families with a clearer sense of prognosis. In many regions, as in the situation already described, having a straightforward method to evaluate collaterals can make a meaningful difference in clinical decision making. This perspective aligns with efforts led by groups such as the Global Stroke Alliance, which highlight the value of practical and low-complexity tools to help reduce gaps in access to reperfusion therapies where they are needed most.

This study has several limitations. First, the sample size is relatively modest and reflects the selective population of early-window large-core stroke patients with complete imaging and outcome data. For this reason, the findings should be interpreted as exploratory. Second, collateral status was assessed using single-phase CTA, which may underestimate delayed collateral filling compared with multiphase imaging techniques (Jansen et al., 2019; Menon et al., 2015). Finally, perfusion mismatch and infarct growth could not be systematically evaluated because follow-up imaging and standardized perfusion parameters were not available for all patients.

Despite these limitations, the consistency of our results across both discharge NIHSS and 90-day mRS strengthens their credibility. Larger multicenter studies are needed to validate these findings, to clarify how collateral status relates to the effects of reperfusion therapies in patients with intermediate or rapid infarct progression, and to determine how collateral-based information can be integrated into treatment algorithms and prognostic models.

## Conclusion

In patients with large-core AIS treated early, collateral grade on baseline CTA was strongly linked to recovery. Even when ASPECTS and core volumes were similar, patients evolved differently depending on their collateral supply. Incorporating a simple collateral assessment into routine evaluation may help refine prognostic stratification and support treatment decisions in this challenging subgroup.

## Data Availability

The raw data supporting the conclusions of this article will be made available by the authors, without undue reservation.
